# Resource-Efficient Use of Modern Processor Architectures For Numerically Solving Cardiac Ionic Cell Models

**DOI:** 10.3389/fphys.2022.904648

**Published:** 2022-06-28

**Authors:** Kristian Gregorius Hustad, Xing Cai

**Affiliations:** ^1^ Simula Research Laboratory, Oslo, Norway; ^2^ Department of Informatics, University of Oslo, Oslo, Norway

**Keywords:** cardiac electrophysiogy, ionic cell models, multicore CPUs, lookup tables (LUTs), SIMD vectorisation

## Abstract

A central component in simulating cardiac electrophysiology is the numerical solution of nonlinear ordinary differential equations, also called cardiac ionic cell models, that describe cross-cell-membrane ion transport. Biophysically detailed cell models often require a considerable amount of computation, including calls to special mathematical functions. This paper systematically studies how to efficiently use modern multicore CPUs for this costly computational task. We start by investigating the code restructurings needed to effectively enable compiler-supported SIMD vectorisation, which is the most important performance booster in this context. It is found that suitable OpenMP directives are sufficient for achieving both vectorisation and parallelisation. We then continue with an evaluation of the performance optimisation technique of using lookup tables. Due to increased challenges for automated vectorisation, the obtainable benefits of lookup tables are dependent on the hardware platforms chosen. Throughout the study, we report detailed time measurements obtained on Intel Xeon, Xeon Phi, AMD Epyc and two ARM processors including Fujitsu A64FX, while attention is also paid to the impact of SIMD vectorisation and lookup tables on the computational accuracy. As a realistic example, the benefits of performance enhancement are demonstrated by a 10^9^-run ensemble on the Oakforest-PACS system, where code restructurings and SIMD vectorisation yield an 84% reduction in computing time, corresponding to 63,270 node hours.

## 1 Introduction

Computer simulation has firmly established itself as an important approach to studying cardiac electrophysiology, see e.g. Vigmond et al. (2009); Trayanova (2011). One essential component of any heart simulator is the computation of the total transmembrane *ionic current density*, conventionally denoted by *I*_ion_. The importance of *I*_ion_ is due to its close interaction with the *transmembrane potential* *v*, i.e., the difference between the intra- and extracellular potentials. A coordinated evolvement of $v\left(\vec{x},t\right)$ in space and time is a prerequisite for the proper functioning of the heart. Physiologically, *I*_ion_ is intricately determined by various transmembrane currents through ionic channels, pumps and exchangers, even subcellular calcium handling. Thus, mathematical modeling of *I*_ion_ is challenging and still remains an active research field. Many cell models have been developed over the years, where two examples of widely used cell models are the ten Tusscher–Panfilov model (see ten Tusscher and Panfilov (2006)) and the Grandi–Pasqualini–Bers model (see Grandi et al. (2010)).

The majority of the cell models take the form of a system of nonlinear first-order ordinary differential equations (ODEs) with initial conditions:

$$\frac{d\vec{s}\left(t\right)}{dt}=\vec{f}\left(t,\vec{s}\left(t\right)\right),\;\vec{s}\left(0\right)={\vec{s}}_{0},$$ (1)

where $\vec{s}\left(t\right)$ is a vector of so-called state variables including the transmembrane potential itself, a set of individual ionic concentrations, and a set of gating variables, see e.g. Alonso et al. (2016) for a review. The evolution of $\vec{s}\left(t\right)$ determines the total transmembrane ionic current density *I*_ion_, which in turn contributes to the dynamic change of *v*. The particular ODE inside the system in Eq. (1) that describes the dynamics of *v* has its simplest form as follows:

$$\frac{dv}{dt}=-\frac{1}{C_{m}}I_{\text{ion}},$$ (2)

where *C*_*m*_ denotes the membrane capacitance. If electrophysiology is simulated over a cardiac tissue or the entire heart, then Eq. (2) can be incorporated into a partial differential equation (PDE) of the reaction-diffusion type, such as in the monodomain and bidomain models, see e.g. Colli Franzone et al. (2014).

### 1.1 Need for Huge Amounts of Computation

Many of the right-hand side functions in Eq. (1), i.e., *f*_1_, *f*_2_, …, *f*_*N*_, where *N* denotes the number of state variables of a cell model, are nonlinear and involve special mathematical functions such as the exponential, logarithmic and power functions. For example, the ten Tusscher–Panfilov model [see ten Tusscher and Panfilov (2006)] adopts *N* = 19 state variables and the authors’ own C++ source code [see ten Tusscher (2021)] counts 77 calls to the exponential function and 4 calls to the logarithmic function. On a computer, these calls to the special mathematical functions will be translated into a large number of basic floating-point operations (FLOPs). For example, profiling tools have revealed that typical compilers will generate in total around 1500 FLOPs each time the 19 right-hand side functions of the ten Tusscher–Panfilov model are evaluated on a computer (see Section 3.2).

ODE computations can use substantial time of a heart simulator. Inside a monodomain or bidomain simulator of a cardiac tissue or an entire heart, an ODE system of form Eq. (1) exists “everywhere”, i.e., with the same spatial resolution as for the intra- and extracellular potential fields. For the latest simulation strategy based on the EMI (extracellular-membrane-intracellular) approach, see e.g., Tveito et al. (2017); Jæger et al. (2021a), a high spatial resolution is still needed to resolve the cell membrane surfaces, resulting in considerable computational effort needed to solve the individual ODE systems. Multiple studies have investigated how simulations using the monodomain model or the bidomain model can be scaled to thousands of compute nodes [see e.g., Niederer et al. (2011); Mirin et al. (2012); Colli Franzone et al. (2018)]. Operator splitting is typically used with the monodomain, bidomain, and EMI models such that the non-linear ODE part is decoupled from the linear PDE part [see Clayton et al. (2011); Tveito et al. (2017)]. Thus, the performance of the ODE part, which does not require any communication, may be studied independently of the PDE part. For whole-heart simulations using the monodomain or bidomain model with reasonably accurate meshes, the number of ODE systems is in the millions, even ranging as high as 370 million [see Mirin et al. (2012)], whereas the time step is typically limited to around 25 µs [see Niederer et al. (2011)]. In other words, 40,000 time steps must be solved for each second of simulated time.

Besides the above *simulation scenario*, an *ensemble scenario* can also require solving many instances of a cell model. This is needed to study the sensitivity of a cell model with respect to its internal parameters, or to fit the model parameters with real-word cellular measurements [see e.g., Jæger et al. (2021b)]. The number of instances can easily be colossal, if the number of parameters of interest is large and/or the resolution needed to study each parameter is high.

No matter which scenario, when the required temporal/spatial/parameter resolution is high, there arises the need for numerically solving a large number of ODE system instances over a large number of time steps. This can lead to a gargantuan amount of computing time even on a supercomputer. The present paper thus aims to investigate how the modern multicore CPU architectures can be efficiently used for this purpose.

### 1.2 Need for Effective Use of Modern Processor Architectures

The primary design goal of a modern multicore CPU is to execute FLOPs fast. This is in principle a good match with numerically solving cardiac ionic cell models, which typically have a high computational intensity, i.e., the number of FLOPs executed per byte of memory traffic. Effective utilisation of the floating-point capability of a multicore CPU requires employing all the processor cores while each delivers a sizeable portion of its theoretical peak floating-point performance. Achieving the latter is not straightforward, because it requires each processor core to execute, most of the time, in a single-instruction-multiple-data (SIMD) style. The individual ODE system instances, in both simulation and ensemble scenarios, can be computed independently and thus readily offer parallelisation across the processor cores. However, inappropriate data structures, memory access patterns and/or code structure can seriously limit or even prohibit SIMD vectorisation. This important topic will be addressed in Section 2.3.

Executing FLOPs using SIMD vectorisation alone does not necessarily lead to the best computing speed. Another concern is the necessity of the FLOPs. Modern compilers are good at common subexpression elimination, thus avoiding unnecessary repetitions of FLOPs, but they are unable to decide the most economical way of evaluating the special mathematical functions. A classical method is to pre-evaluate a costly function for a certain value range and resolution, and store these pre-computed values in a *lookup table*. Later evaluations of the function are then replaced by reading (approximate) values from the table. The number of arithmetic operations is reduced at the cost of extra memory usage by the lookup table itself and extra memory traffic due to repeatedly accessing the lookup table. Moreover, using lookup tables may prohibit a compiler from vectorising the other parts of the computation. Section 2.4 will thus discuss the considerations and programming details about lookup tables.

The contribution of this paper is not about devising new ODE solvers with lower algorithmic complexity, higher accuracy or better stability. Instead, our approach to getting fast computing speed is rooted in a resource-efficient usage of modern multicore processor architectures. We discuss the code restructurings that are needed to help modern compilers automatically enable SIMD vectorisation. The speed improvement due to vectorisation is thoroughly investigated by both time measurements and profiling. To our knowledge, these aspects have not been systematically studied in the literature.

Another novelty of this paper is a deep dive into the pros and cons of using lookup tables, where we also study some related programming nuances. Although a number of choices concerning the trade-off between accuracy and speed may be considered when using lookup tables, we have devoted our attention to the programming details. We report accuracy results to verify the correctness of our implementation and contrast with the error associated with the use of SIMD vectorisation.

The ODE models used in this paper for performance study in Section 3 are realistic cardiac ionic cell models (see Table 1), whereas we have only adopted the simplest ODE solvers. The rationale is that more sophisticated ODE solvers often use simple ODE solvers as the building blocks. Thus, a thorough understanding of how to obtain hardware resource efficiency for simple ODE solvers is readily extended to the wealth of advanced ODE solvers.

**TABLE 1:** Cell models used in the numerical experiments of this paper. The “FLOPs” column lists the number of floating-point operations required to compute a single time step for a naïve implementation using the Forward Euler scheme. Section 3.2 describes how performance counters were used to obtain the operation counts.

Model	Name	State variables	FLOPs	References
ten Tusscher–Panfilov (2006)	TP06	19	1500	ten Tusscher and Panfilov (2006)
Jæger–Tveito (2021)	JT21	25	1322	Jæger et al. (2020, 2021a)
Grandi–Pasqualini–Bers (2010)	GPB	39	2149	Grandi et al. (2010)

The remainder of this paper is organised as follows. Section 2.1 briefly explains the basic steps of implementing ODE solvers, including directive-based parallelisation. Section 2.2 points out the inefficiency of naïvely implemented ODE solvers. Section 2.3 carefully examines the topic of SIMD vectorisation on modern multicore processors. Section 2.4 is devoted to the details of using lookup tables as an alternative to getting fast computing speed. Section 2.5 demonstrates how SIMD vectorisation and lookup tables can be combined. Thereafter, Section 3 contains an extensive set of numerical experiments about the benefits due to, respectively, SIMD vectorisation and lookup tables. The topic of accuracy also receives close attention in Section 3. Finally, Section 4 comments on the related work and provides some concluding remarks.

## 2 SIMD Vectorisation and Lookup Tables for Delivering Performance

As argued in Section 1.1, huge amounts of computation may arise from numerically solving many instances of a cell model, in both simulation and ensemble scenarios. At the same time, as discussed in Section 1.2, utilising the computational potential of modern processors can be non-trivial. This section thus aims to investigate two strategies for enhancing the performance of typical solvers of a cell model, specifically, use of SIMD vectorisation and lookup tables. We will start with explaining the basic steps of implementing an ODE solver, for the purpose of setting the programming scene needed to dive into the two strategies.

### 2.1 Basic Steps of Implementing an ODE Solver

The basic steps of implementing an ODE solver are largely generic, such that automated code generation can allow an easy plug-and-play of the solution strategy and cell model, while keeping manual coding to a minimum level. We will use as an illustrating example the simplest ODE solver, namely, the forward Euler method. This choice is motivated by both its simplicity and its relevance as building blocks in many advanced ODE solvers. Simplicity is also the reason for choosing, later in this section, the FitzHugh-Nagumo cell model [see FitzHugh (1961); Nagumo et al. (1962)] that has only two state variables, for the ease of presentation. We remark that the same (automated) programming process applies to other ODE solvers and cell models.

Specifically, to numerically solve a system of ODEs in the form of Eq. (1), the computational work per time step of the forward Euler (FE) method is as follows:

$$\vec{s}\left(t_{\ell +1}\right)\approx \vec{s}\left(t_{\ell }\right)+\Delta t\cdot \vec{f}\left(t_{\ell },\vec{s}\left(t_{\ell }\right)\right).$$ (3)

This simple numerical scheme only requires evaluating the right-hand functions $\vec{f}$ with the latest state variables. The downside of FE is that it may require a very small time step size Δ*t* = *t*_*ℓ*+1_ − *t*_*ℓ*_, and thereby a tremendous number of steps, to produce a stable solution of a stiff ODE system.

#### 2.1.1 Algorithmic Skeletons

The algorithmic skeleton for solving individual ODE system instances in a simulation scenario will differ from that in an ensemble scenario (see Section 1.1). Suppose the computational work of an ODE solver (e.g., FE) per time step is coded as a subroutine named compute_ODE_step $\left(\vec{s},t,\Delta t\right)$ , these two algorithmic skeletons can be found, respectively, in Algorithm 1 and Algorithm 2. Moreover, a realistic simulation scenario can also have a PDE component per time step, due to operator splitting used for solving e.g. the monodomain or bidomain equations. This is shown in Algorithm 3 where the *v* values from all the cells are jointly updated per time step additionally to accommodate the PDE contribution.

Algorithm 1 Simple skeleton for the simulation scenario (outer loop over time).

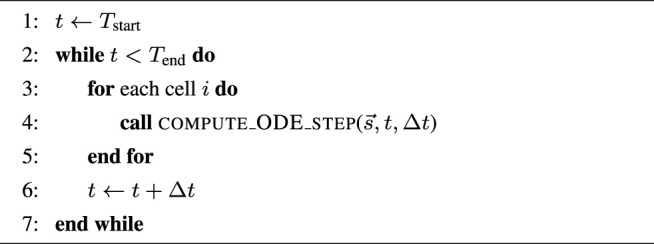

Algorithm 2 Simple skeleton for the ensemble scenario (outer loop over cells).

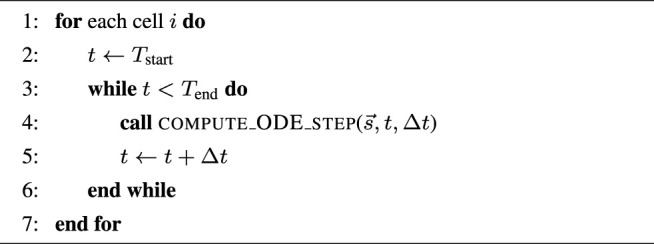

Algorithm 3 Operator-splitting skeleton for the simulation scenario (outer loop over time).

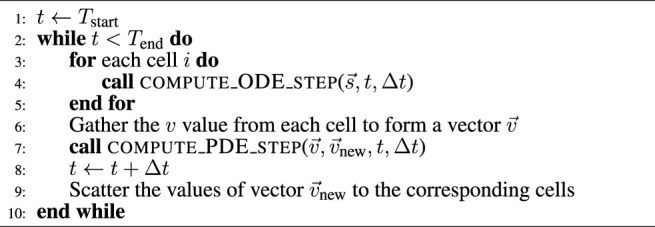

#### 2.1.2 Automated Code Generation

Mathematical models of the ionic current density are typically very complex, involving many parameters and internal variables. Manual coding of compute_ODE_step, which is needed in any of the algorithmic skeletons, can therefore be nontrivial and error-prone. Here, two factors support an approach of automated code generation. First, most ODE solution strategies are generic and independent of a specific cell model. Second, the research community has developed several domain-specific standards to facilitate sharing of the existing cell models, thus offering standardised input to automated code generators. One such open standard is the CellML language [see Cuellar et al. (2003)] based on XML. The benefits of automation include avoidance of human programming errors, a flexible choice of the programming language for the generated code, and easy experimentation with different cell models.

As an example, we will show in Listing 2 a piece of auto-generated code that implements a single FE step for the two-variable FitzHugh-Nagumo (FHN) cell model [see FitzHugh (1961); Nagumo et al. (1962)]:

$$\begin{array}{cc} \frac{dv}{dt} & =v\left(v-\alpha \right)\left(1-v\right)-w+I_{\text{stim}},\\ \frac{dw}{dt} & =\varepsilon \left(v-\gamma w\right), \end{array}\;I_{\text{stim}}=\left\{\begin{array}{cc} -80\; & 0\leq t\leq 0.5,\\ 0\; & otherwise, \end{array}\right.$$ (4)

where *v* and *w* are state variables, and *α*, *ɛ* and *γ* are model parameters. Correspondingly, Listing 1 contains two assisting enum types that are used instead of integer literals when indexing arrays in order to improve readability. The FHN model is used in code listings in this section due to its simplicity. The results presented in Section 3 use the more realistic models listed in Table 1.

Listing 1: Auto-generated enum declarations for the FHN model.

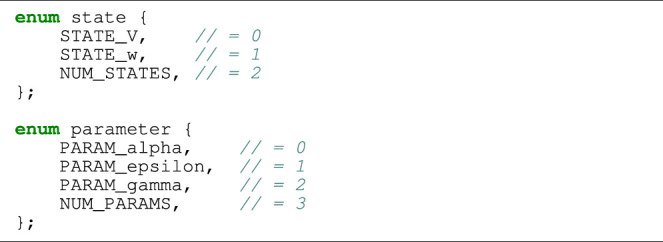

Listing 2: Auto-generated code of one FE step applied to the FHN model.

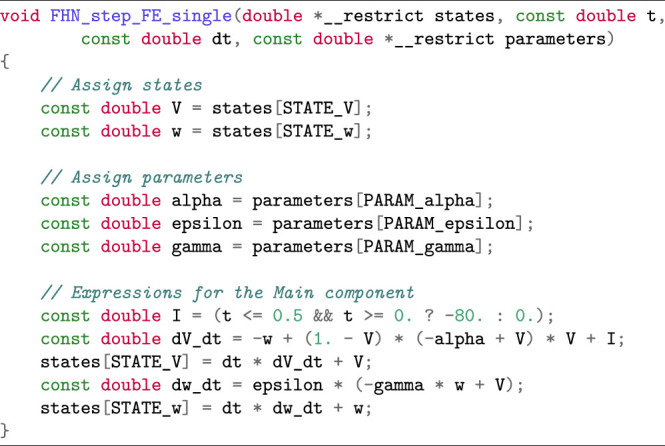

The C code in Listing 2 is auto-generated by Gotran [see Hake et al. (2020)]: a code generation framework for cell models and the associated ODE solvers. The input format of the FHN model can be found at the CellML website [see CellML (2022)].

#### 2.1.3 Shared-Memory Parallelisation Using OpenMP

The need for parallelisation arises when the number of ODE system instances involved in a simulation or ensemble scenario is large. For both cases, parallelisation is straightforward because the ODE system instances can be computed independently. An automated code generator, such as Gotran, can easily create a subroutine that uses OpenMP directives for this purpose. Listing 3 is such an example, which loops over a collection of cells and invokes FHN_step_FE_single (implemented in Listing 2) for each cell. The code in Listing 3 is typically used in a simulation scenario, wrapped within an outer loop over time.

Listing 3: Example of OpenMP parallelisation (simulation scenario).

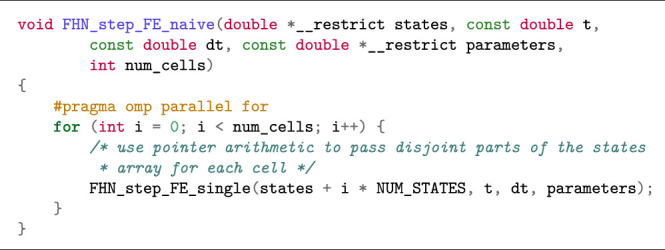

### 2.2 Issues of Inefficiency

The auto-generated code shown in Listings 2 and 3 can be readily used in any simulation scenario. A corresponding version of Listing 3 for an ensemble scenario can also easily be auto-generated. Switching to another cell model and/or a different explicit ODE solver will in general pose no challenge. Thanks to the automated insertion of OpenMP directives, the generated code can use all the CPU cores of a shared-memory system. Listings 2 and 3 can also be used without change inside a distributed-memory parallel monodomain or bidomain simulator. (Here, we assume that each MPI process is assigned with a partition of the cells.)

However, we can only label the above auto-generated code as naïve, because the obtained performance will be considerably lower than the ideally achievable level. The main reason is the inability of compilers to enable SIMD vectorisation for this code. Specifically, the first argument to function FHN_step_FE_single in Listing 2 assumes that the *N* state variables for each cell are stored contiguously in memory. This means that the state variables of all the cells are stored logically as an “array of structs”, as used by Listing 3. Although such a data structure makes sense by grouping the state variables of each cell, the downside is that compiler-supported SIMD vectorisation will fail completely. Code restructuring needed for auto-vectorisation will be addressed in Section 2.3, whereas the potential performance benefits of using lookup tables will be the topic of Section 2.4.

### 2.3 SIMD Vectorisation

#### 2.3.1 Computing with Vectors

Modern CPUs use special registers and instructions for SIMD vectorised operations. For example, the AVX-512 vector instruction set provides 512-bit vectors, so that eight double-precision (64-bit) floating-point numbers may be stored together in a vector register, and arithmetic operations such as addition and multiplication can be performed simultaneously to all the numbers stored in these vectors. To efficiently read and store vector registers, the content of a vector should lie *contiguously* and *aligned* in memory. The latter means that the start address of the vector in memory is a multiple of the vector width.

Conceptually, simultaneous solution of multiple instances of the same cell model suits perfectly for SIMD vectorisation. This is because the identical computation takes place in the different cells, i.e., the same operations are applied to different values. The rare situation of conditional branching (e.g., the outcome of an if test depends on the actual value of a state variable) can also be vectorised through masking. In the following, we will discuss how to restructure the auto-generated naïve code, so that compilers can automatically carry out the SIMD vectorisation, by using suitable compiler options/hints and vectorised math libraries.

#### 2.3.2 Restructuring for Optimal Memory Layout

As discussed in Section 2.2, the auto-generated naïve code (as shown in Listings 2 and 3) adopts a natural but vectorisation-unfriendly data structure, where the state variables of each cell are stored contiguously in memory. For effective use of the vector registers, a vectorisation-friendly data structure should let the same state variable from all the cells be stored contiguously. The entire data structure thus has the layout of a “struct of arrays”. To guarantee memory alignment, each state-variable array may need to be padded. Suppose the number of cells is *C*, the number of no-use 64-bit values padded at the end of each array can be calculated as 8 - modulo(C,8) for the case of 512-bit vector width. In practice, all the state-variable arrays (with padding) are concatenated into a very long 1D array. This can be seen in Listing 4.

#### 2.3.3 Compiler-Supported Auto Vectorisation for the Simulation Scenario

When the memory-related code restructuring is done, SIMD vectorisation can be automatically enabled by a compiler. There are multiple ways of providing vectorisation hints to a C compiler, but we will focus on the simd construct of OpenMP as it is supported by all the major compilers. (The code examples given in this paper require OpenMP version 4.5 or newer.) In Listing 3, we used the compiler directive of #pragma omp parallel for to parallelise the for loop. Listing 4 shows a modified version based on a restructured SIMD-friendly data layout, where we have also added the necessary compiler hints to enable auto-vectorisation. Specifically, the additional simd clause suggests to the compiler that multiple iterations of the loop could be computed together as a vector.

The simd clause may be followed by additional clauses: The simdlen clause specifies the preferred number of lanes per SIMD vector. In the following code listings, we assume that the user has defined the constant VECTOR_LENGTH which is passed as the argument to simdlen. The aligned clause can be used to provide information about the alignment of arrays, so that the compiler can employ aligned vector load/store instructions.

When using the Clang compiler, however, specifying only OpenMP pragmas does not lead to successful vectorisation, because Clang is unable to prove that vectorisation can safely be applied. We therefore specify an additional Clang-specific pragma, see Listing 4, where we instruct the compiler to assume memory safety, relieving Clang of the requirement to prove that there are no overlapping memory accesses. For the sake of brevity, we only show the vectorisation hints based on OpenMP in the remaining listings.

Listing 4: Step function for the FitzHugh-Nagumo model (simulation scenario) with SIMD-friendly data layout restructuring.

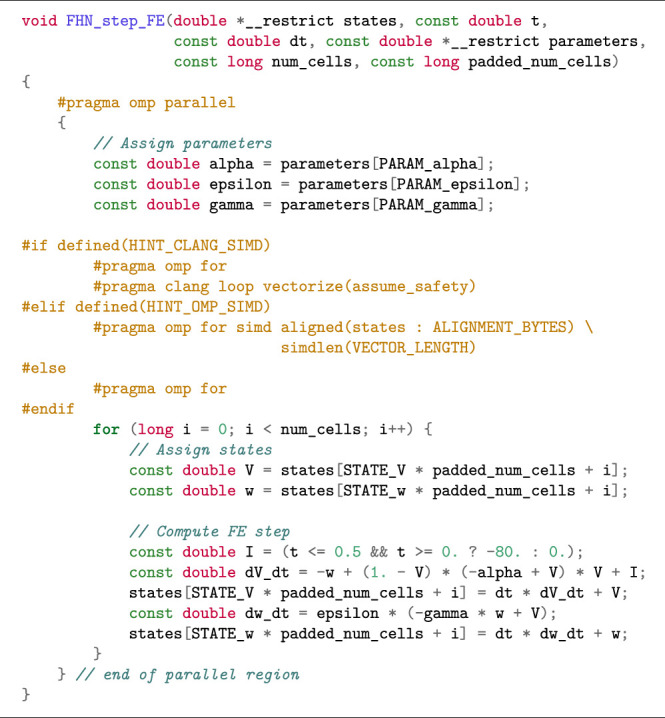

#### 2.3.4 Compiler-Supported Auto Vectorisation for the Ensemble Scenario

The simd OpenMP clause works best when applied to the inner loop. In case the outer loop is over the cells, vectorisation becomes more complicated as the compiler will have to perform *outer loop vectorisation*. Listing 5 shows how to restructure the code in the ensemble scenario to enable auto vectorisation. When the loops are structured in this manner with cells in the outer loop and time in the inner loop (we refer to this loop structure as Cell–Time), we minimise memory traffic, as the parameters and state variables can easily fit in cache between two time steps. Furthermore, expressions that are not a function of the state variables or time will not change between time steps and can therefore be reused such that the total amount of computation is reduced. Since the number of iterations in the inner loop over time (i.e., the number of time steps) is the same for all cells, outer loop vectorisation can safely be applied in this scenario. However, we observed that only the Intel compiler was able to perform vectorisation for the function in Listing 5. We therefore investigated two alternative loop structures in order to achieve vectorisation with the other compilers.

Listing 6 uses a Time–Cell loop structure similar to the simulation scenario, with the difference being that the parameters are no longer shared between all cells. This loop structure can lead to very high memory traffic, because all parameters must be read and all state variables read and written for each inner iteration in the loop. The three code blocks following the comments “Assign parameters”, “Assign states”, and “Compute FE step” are the same as in Listing 5 and were omitted for brevity.

If we solve the model for smaller batches of cells, we facilitate caching of both arrays and reduce the memory traffic. Listing 7 shows a Cell–Time–Cell loop structure where the outermost loop divides the work into batches that are mapped to different OpenMP threads with the “parallel for” directive, and then the middle loop iterates over time, whereas the innermost loop iterates over the elements in a batch. We should choose the batch size to be a multiple of the hardware SIMD vector length, and the batch size is here controlled *via* a compile-time defined constant VECTOR_LENGTH. In practice, all vector lengths are a power of 2, so 2^5^ = 32 would be a reasonable choice of batch size that would work well on any CPU. Note that some extra bookkeeping is needed to handle the case where the total number of cells does not evenly divide the batch size. The performance of the different loop structures is discussed in Section 3.4.

Listing 5: Cell–Time loop structure in FE solution of the FitzHugh-Nagumo model (ensemble scenario) with SIMD-friendly data layout restructuring.

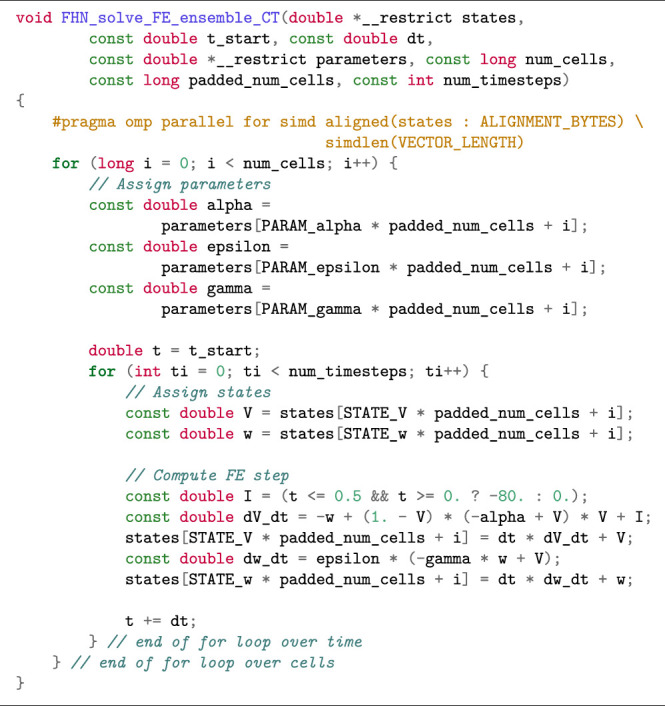

Listing 6: Time–Cell loop structure in FE solution of the FitzHugh-Nagumo model (ensemble scenario) with SIMD-friendly data layout restructuring.

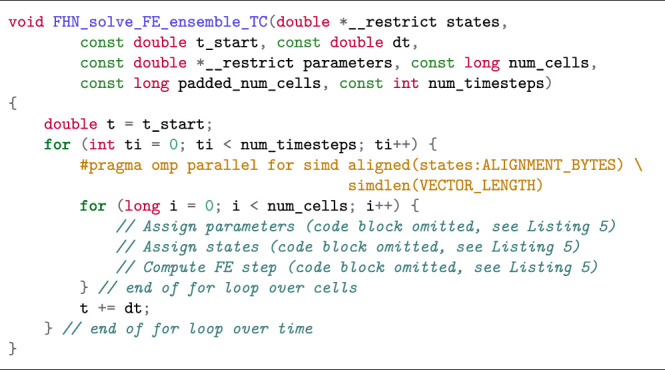

Listing 7: Cell–Time–Cell loop structure in FE solution of the FitzHugh-Nagumo model (ensemble scenario).

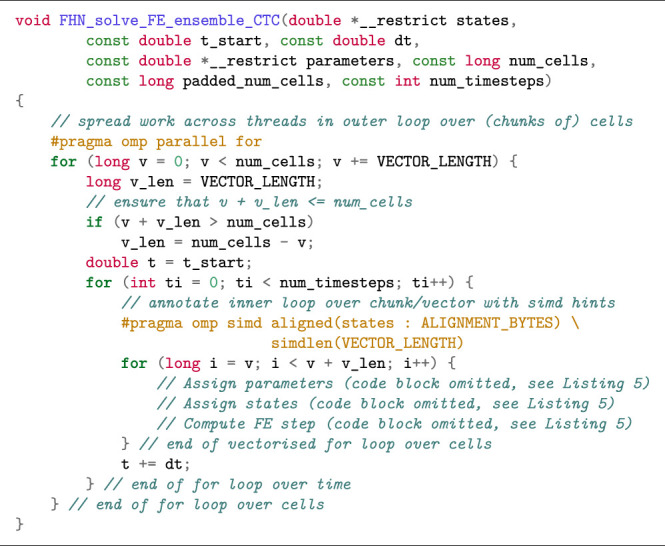

#### 2.3.5 Using Vectorised Math Libraries

The cell models often involve calls to the exponential function (exp), the logarithmic function (log), and the power function (pow). The expm1 function is also relevant in the context of some numerical schemes that require evaluating *e* ^*x*^ − 1 where *x* may be close to zero, in which case exp(x)-1 is prone to rounding errors and expm1(x) produces more accurate results. In the standard C library, these are defined as functions with a scalar input and output, but we need to evaluate these functions on all the elements in a vector simultaneously to achieve SIMD. Fortunately, there are vectorised math libraries that provide C functions that use SIMD instructions to evaluate these math functions for selected vector lengths.

Intel’s Short Vector Math Library (SVML) and the GNU C library, *glibc*, both provide vectorised versions of the relevant math functions for the x86 instruction set. Shibata and Petrogalli [see Shibata and Petrogalli (2020)] developed a vector math library, *SLEEF*, that supports the ARMv8 instruction set in addition to x86. There are also other vector math libraries, but these three libraries all integrate with compiler auto-vectorisation when used with a compatible compiler.

As vectorised implementations of expm1 are not available for all libraries, we have used a preprocessor macro to control whether expm1(x) or (exp(x)-1) is used. expm1(x) is used in the naïve implementations, and (exp(x)-1) is used in all vectorised code unless otherwise stated.

We should note that the libraries vary slightly in the accuracy to which the functions are evaluated, and the instructions used (and thereby the total number of floating point operations) may also vary. Our goal in this study is not to compare the vector math libraries, but they are a necessary component when using auto-vectorisation on cell models that contain calls to math functions. When studying the performance and accuracy of cell models, we will therefore have to consider the influence of the math libraries used. Time measurements of using vectorised math libraries, as well as an accuracy analysis will be provided in Section 3.3.

#### 2.3.6 Explicit Control of the Vector Length

In some instances, the compiler may generate instructions with a shorter vector length than the maximal supported vector length, often resulting in sub-optimal performance. There are mechanisms that allow the user to instruct the compiler to target a specific vector length, but the exact mechanism varies for each compiler. For the Intel compiler on Oakbridge (see Table 2), we observed that 256-bit vectors would be used by default when targeting the Cascade Lake CPU, although the hardware vector length is 512 bits. When we appended simdlen(8) to the simd clause, the compiler would instead use 512-bit instructions (8 SIMD lanes with 64-bit floating-point values). For GCC, the flag -mprefer-vector-width=512 can be used to the same effect.

**TABLE 2:** Hardware specifications (compute node level) of the five target platforms. ISA is an abbreviation of “instruction set architecture”.

Name	CPU	ISA	SIMD width (bits)	Memory	Peak memory bandwidth
Oakforest	1 × Intel Xeon Phi 7250	x86-64	512	16 GiB MCDRAM + 96 GiB DDR4	MCDRAM: > 400 GB/s DDR4: 125 GB/s
	Peak performance: 3 TFLOPS				
Oakbridge	2 × Intel Xeon Platinum 8280	x86-64	512	192 GiB DDR4	281 GB/s
	Peak performance: 4.8 TFLOPS				
Wisteria	1 × Fujitsu A64FX	ARM v8.2-A	512	32 GiB HBM	1024 GB/s
	Peak performance: 3.4 TFLOPS				
Milan	2 × AMD EPYC 7763	x86-64	256	2 TiB DDR4	410 GB/s
	Peak performance: 5.0 TFLOPS				
ThunderX2	2 × Cavium ThunderX2 CN9980	ARM v8.1-A	128	1 TiB DDR4	341 GB/s
	Peak performance: 1.0 TFLOPS				

For the Fujitsu compiler on Wisteria, we pass the flags -msve-vector-bits=512 and -ffj-interleave-loop-insns=4. The second flag instructs the compiler to interleave 4 iterations of the loop such that 4 × 8 cells are processed in parallel in each OpenMP thread. Interleaving loop iterations increases the amount of instruction-level parallelism at the cost of an increased register pressure, which seems to be very beneficial on the A64FX CPU with somewhat higher latencies for arithmetic instructions than the CPUs found in the other systems. We found that interleaving 4 loop iterations yielded the best performance on Wisteria.

### 2.4 Lookup Tables

The rationale for using *lookup tables* is to reduce the amount of computation, by repeatedly referring to tables of pre-computed values. Most cell models contain a number of expressions that are functions of the transmembrane potential *v*, so these expressions may be pre-evaluated for a chosen sampling of the expected *v* values, before the ODE solution procedure. For instance, we may assume *v* ∈ [−100 mV, 50 mV] in the healthy heart, and we can thus pre-evaluate the expressions at equally spaced points in this interval with a resolution of *v*_step_. Expressions that are a function of more than one state variable are usually not considered for using lookup tables, because the memory footprint (and setup cost) of the tables grows exponentially with the number of input variables.

Let *f*(*v*) denote an expression that depends on *v* and assume that a lookup table has been pre-computed for the interval [*v*_min_, *v*_max_] with resolution *v*_step_. When the lookup table is later repeatedly used, the actual *v* values may not coincide with the pre-chosen sampling values. Suppose a particular *v* value lies between two consecutive sampling points: *v*_*a*_ ≤ *v* < *v*_*b*_ = *v*_*a*_ + *v*_step_. The typical strategy is to use a linear interpolation by computing *w_a_* $=\frac{v_{b}-v}{v_{\text{step}}}$ and *w*_*b*_ = 1 − *w*_*a*_, and then use *w*_*a*_ ⋅ *f* (*v*_*a*_) + *w*_*b*_ ⋅ *f* (*v*_*b*_) as the approximation of *f*(*v*). The two values of *f* (*v*_*a*_) and *f* (*v*_*b*_) are fetched from the pre-computed lookup table that is stored in memory. Note that if multiple expressions use the same input variable, the weights *w*_*a*_ and *w*_*b*_ remain the same for all these expressions. For memory efficiency, the pre-evaluated values of these expressions can be collected as a large 2D table where each column corresponds to one expression.

#### 2.4.1 The Rush–Larsen Scheme and Lookup Tables

When solving stiff ODE systems, the forward Euler scheme may require a very small Δ*t* in order to maintain stability. Rush and Larsen [see Rush and Larsen (1978)] proposed the use of an exponential integrator for the gate variables, which are governed by quasi-linear equations on the form:

$$\frac{dw}{dt}=\frac{w_{\infty }\left(v\right)-w}{\tau_{w}\left(v\right)},$$ (5)

where $w_{\infty }$ and *τ*_*w*_ are functions of the transmembrane potential. Let *v*_*i*_ denote the latest computed transmembrane potential. If *v* is assumed to be constant when updating *w*, Eq. (5) becomes a linear ODE with an analytical solution:

$$w\left(t_{i}+\Delta t\right)=\left[w\left(t_{i}\right)-w_{\infty }\left(v_{i}\right)\right]e^{-\Delta t/\tau_{w}\left(v_{i}\right)}+w_{\infty }\left(v_{i}\right).$$ (6)

The Rush–Larsen scheme (RL) applies Eq. (6) to all the gating equations, whereas the FE scheme in Eq. (3) is used for the remaining equations.

When using lookup tables in the context of RL, it is convenient to rewrite Eq. (6) on the form:

$$w\left(t_{i}+\Delta t\right)=a\left(v_{i}\right)\cdot w\left(t_{i}\right)+b\left(v_{i}\right),$$ (7)

where *a* (*v*_*i*_) and *b* (*v*_*i*_) are two pre-tabulated expressions:

$$a\left(v_{i}\right)=e^{-\Delta t/\tau_{w}\left(v_{i}\right)},$$ (8)

$$b\left(v_{i}\right)=-w_{\infty }\left(v_{i}\right)\left[e^{-\Delta t/\tau_{w}\left(v_{i}\right)}-1\right].$$ (9)

Sundnes et al. [see Sundnes et al. (2009)] showed that the RL scheme can be generalised to equations that are not quasi-linear by performing an additional linearisation step. This leads to a first-order accurate generalised Rush–Larsen scheme, which we will simply refer to as GRL1. Both RL and GRL1 schemes will be used in the numerical experiments later.

#### 2.4.2 Memory Layout of a Multi-Expression Lookup Table

A lookup table containing *M* expressions evaluated at *S* points, can be represented as an *S* × *M* array in memory. This choice of memory layout suits well for the row-major storage scheme used by the C programming language. To find linear interpolations of the *M* expressions with the same input variable, we need to extract two consecutively tabulated values for each expression. Accessing two consecutive rows of the tabulated values (when the table is *S* × *M*) is much more efficient than accessing two columns that are not contiguous in memory (if the table is *M* × *S*).

We have implemented the ten Tusscher–Panfilov (TP06) model [see ten Tusscher and Panfilov (2006)] using lookup tables. Our selection of tabulated expressions is based on the implementation found in the openCARP cardiac simulator [see Plank et al. (2021)], which also makes use of the RL scheme. In total 12 out of the 19 state variables are gating variables that can be solved with Eq. (7). Eleven of the gating variables are functions of *v*, and the last one is a function of Ca_ss_ (free diadic subspace calcium concentration). We have therefore created one lookup table for the 11 expressions related to *v* and another table for Ca_ss_. The *v* table also contains 6 expressions that appear on the right hand side of the equations for the non-gating variables.

### 2.5 Combining SIMD Vectorisation and Lookup Tables

Reconciling the scattered memory access patterns arising from the use of lookup tables with the need for contiguous memory accesses for effective use of SIMD vectorisation is non-trivial. We propose a partitioned method for the TP06 model where lookup tables are used for the 12 gating variables, and the non-gating variables are computed using SIMD vectorisation. The rationale for this partitioned method is that computing the gating equations using lookup tables is very effective. Specifically, updating each gating variable requires only eight FLOPs: two FLOPs for Eq. (7), in addition to the three FLOPs required for the linear interpolation of each of the two pre-tabulated expressions (*a* and *b*). Listing 8 shows the code skeleton with two inner loops.

Listing 8: Combined use of SIMD vectorisation and lookup tables when solving the ten Tusscher–Panfilov model with the RL scheme (simulation scenario).

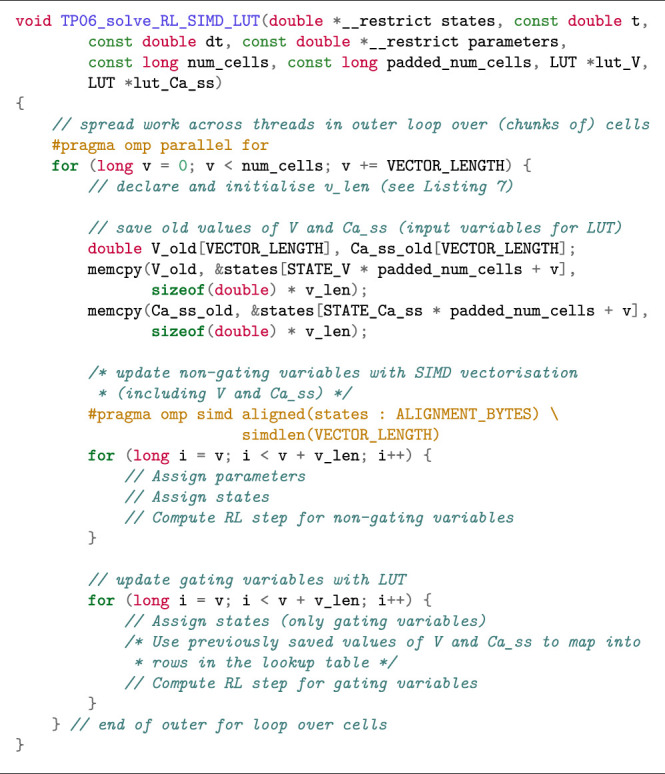

The complete source code for the experiments in this paper is available online at https://github.com/KGHustad/cell-model-cpu-code.

## 3 Experiments and Performance Measurements

### 3.1 Overview of Hardware Testbeds

As our aim in this paper is to compare different optimisation strategies for a wide range of CPU architectures, we have used five different hardware testbeds. The first three testbeds are using the supercomputers Oakforest-PACS, Oakbridge-CX and Wisteria-O operated by the Information Technology Center at the University of Tokyo, whereas the “Milan” and “ThunderX2” testbeds are part of the eX3 infrastructure hosted at Simula Research Laboratory. Each testbed consists of a single compute node with one or two multicore CPUs. An overview of the CPU and memory specifications for the testbeds is given in Table 2.

Table 3 lists the compiler flags we used to enable auto-vectorisation. Since Listing 4 makes use of preprocessor conditionals, we have provided additional flags to define constants controlling which code path is compiled. With ARMClang and the Fujitsu compiler (running in Clang mode) we pass the flag -DHINT_CLANG_SIMD, and with the other compilers we pass the flag -DHINT_OMP_SIMD).

**TABLE 3:** Compiler flags used to enable auto-vectorisation.

Compiler	Version	System	Flags
ARMClang	21.0	ThunderX2	-O3 -fopenmp -ffast-math -fsimdmath -fno-math-errno
Fujitsu	4.7.0	Wisteria	-Nclang -Ofast -fopenmp
GCC	11.1.0	Milan	-O3 -fopenmp -ffast-math -march=native
Intel	19.1.3.304	Oakbridge/ Oakforest	-O3 -qopenmp -fp-model fast=2 -march=native

### 3.2 Counting Floating-Point Operations with Performance Counters

Some CPU architectures provide performance counters that enable the programmer to count the number of floating-point instructions executed for each vector length. The set of performance counters available is highly architecture-dependent, and we will limit our discussion here to the Intel Cascade Lake CPU architecture found on the “Oakbridge” system in Table 2. Listing 9 demonstrates how the perf command in Linux can be used to count number of floating-point operations (FLOPs). Note that the performance counters must be multiplied by the number of SIMD lanes and summed up in order to obtain the total FLOP count. Written out, the total number of floating-point operations is computed as

$$\begin{array}{ll} \text{floating}-\text{point operations} & =8\cdot FP\text{\_}\mathrm{ARITH\_INST\_RETIRED.512B\_PACKED\_DOUBLE}\\ & +4\cdot FP\text{\_}\mathrm{ARITH\_INST\_RETIRED.256B\_PACKED\_DOUBLE}\\ & +2\cdot FP\text{\_}\mathrm{ARITH\_INST\_RETIRED.128B\_PACKED\_DOUBLE}\\ & +1\cdot FP\text{\_}\mathrm{ARITH\_INST\_RETIRED.SCALAR\_DOUBLE}. \end{array}$$

Listing 9: Command used to count number of floating point operations on Intel Cascade Lake CPUs.

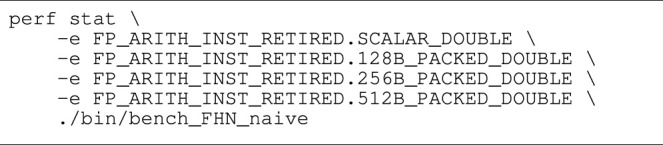

In Table 1 we have used performance counters to count the number of floating-point operations required to solve a single step with a naïve FE implementation for each of the three cell models that are used in this section.

### 3.3 Speed and Accuracy of Vectorised Math Libraries

We measured the performance of selected scalar and vectorised math functions for each of the testbeds. The math function is called for every element in an array of length 30,000, and then this is repeated 20,000 times in an outer loop. We annotate the inner loop with #pragma omp for simd to enable vectorisation via OpenMP. We have selected the exp(), expm1(), log(), pow() functions for this benchmark, as these are the only math functions that are used when solving the cell models in this paper. The benchmark is single-threaded, and the number of function evaluations per second is reported in Table 4.

**TABLE 4:** Single-threaded performance of scalar and vectorised math library calls. The units for the Scalar and SIMD columns is millions of function evaluations per second.

System	Function	Scalar	SIMD	Speedup
Oakbridge	exp	261.0	760.6	2.91
Oakbridge	expm1	167.4	648.8	3.88
Oakbridge	log	202.4	663.6	3.28
Oakbridge	pow	83.4	427.1	5.12
Oakforest	exp	43.9	260.0	5.92
Oakforest	expm1	23.7	223.4	9.44
Oakforest	log	38.5	247.1	6.41
Oakforest	pow	13.9	103.9	7.50
Milan	exp	91.7	743.6	8.11
Milan	expm1	151.8	138.3	0.91
Milan	log	73.9	583.6	7.89
Milan	pow	26.8	159.7	5.96
Wisteria	exp	85.6	633.1	7.40
Wisteria	expm1	25.3	24.3	0.96
Wisteria	log	79.1	534.7	6.76
Wisteria	pow	16.4	113.4	6.92
ThunderX2	exp	88.4	123.0	1.39
ThunderX2	expm1	45.6	27.1	0.59
ThunderX2	log	64.8	102.9	1.59
ThunderX2	pow	25.6	22.6	0.88

The Intel-based systems (Oakbridge and Oakforest) both see a speedup when using SIMD, and although the speedup is more pronounced on Oakforest, the absolute performance is 2.5–4 times higher on Oakbridge, despite having only 1.6 times the theoretical peak performance. The last x86 system, Milan, achieves speedup that exceeds expectations, considering that its 256-bit vector length would allow it to perform arithmetic at 4 times the rate of a scalar implementation. The theoretical peak performance of Milan and Oakbridge are comparable, and it seems likely that the high speedup is explained by the scalar implementations performing poorly on Milan. As there is no vectorised implementation of expm1() in libmvec version 2.27, we see no speedup for that function.

On Wisteria, we observe a reasonable 6–8 times speedup for all functions except expm1(). ThunderX2 achieves a modest speedup only for the exp() and log() functions, whereas the vectorised expm1() function sees a substantial degradation in performance.

We also measured the accuracy of the vectorised math libraries by calling each function with 1 million different input values, and then comparing the result with reference solutions with the MPFR library [see Fousse et al. (2007)] computed using 120 bits of accuracy. Table 5 reports the maximum error in units of least precision (ULPs). If the true, infinite-precision value is located on the midpoint between two floating-point values of similar magnitude, the error would be 0.5 ULPs. The input values were generated by interpreting randomly generated bytes as a double-precision floats, and then we discarded values outside the desired domain (the input domains are listed in Table 5) and subnormal values (that are too small to represent in full precision). For the pow() function, we also omit values where the answer would have been subnormal.

**TABLE 5:** Maximum error of vectorised math library calls when evaluating input values in the prescribed ranges. The error is reported in units of least precision (ULPs).

Function (value range) System	exp (−700, 700)	expm1 (−700, 700)	log (10^–300^, 10,^*300*^)	pow (—30, 30) × (—30, 30)
Oakbridge	2.623	2.753	1.496	0.998
Oakforest	1.471	2.008	1.276	1.035
Milan	2.623	0.735	1.343	0.998
Wisteria	1.923	0.753	1.343	1.62×10^13^
ThunderX2	2.313	0.992	1.883	0.998

pow() on Wisteria has very poor accuracy for two input values where the correct answers (6.11 × 10^–308^, 2.48 × 10^–307^) are small but not subnormal (as they are greater than 2.23 × 10^–308^). If those two values are ignored, the maximum error was 36.206 ULPs, which is still significantly greater than the other systems. The remaining errors reported in Table 5 are all below 3 ULPs, which should be well within the accuracy requirements for the solution of cell models.

### 3.4 Performance of Vectorised ODE Solvers

In our performance measurements of the ODE solver, we use the throughput metric “cell steps per second”, which is simply defined as

$$\text{cell steps per second}=\frac{\text{number of cells}\times \text{number of time steps}}{\text{solution time in seconds}}.$$ (10)

The advantage of a such a throughput metric is that it simplifies the comparison of results with differing numbers of cells or time steps, and it can easily be used to estimate the solution time for a problem with a given number of cells and time steps.

Table 6 and Table 7 show the single-threaded and multi-threaded performance of the naïve and auto-vectorised implementations using the FE scheme, where we have used it to solve three realistic ODE models as shown in Table 1. We set OMP_NUM_THREADS=1 in the environment when measuring single-threaded performance, and we set it to the number of logical cores when measuring the multi-threaded performance. The “SoA” column uses a “struct of arrays” memory layout, as discussed in Section 2.3.2, but it does not provide any SIMD hints to the compiler. The “SIMD” column adds SIMD hints to the SoA implementation. We also report speedup factors comparing the SIMD implementation to both the naïve and the SoA implementation. The CPU clock speed is typically somewhat lower when executing vector instructions than when execution scalar instructions, especially when all cores are under heavy load, and this is one of the reasons why the speedup is generally higher in the single-threaded case. The other reason would be that we are more likely to encounter a memory bandwidth bottleneck when using all threads. For the JT21 model on the ThunderX2, we see a pronounced reduction in performance for the SoA implementation compared to the naïve implementation, but the other system–model combinations generally show a slight improvement for SoA over naïve.

**TABLE 6:** Single-threaded performance of naïve and auto-vectorised implementations. The FE scheme is used; *C* = 11688851 cells. “SoA” refers to the “struct of arrays” memory layout discussed in Section 2.3.2.

System	Model	Throughput $\left(10^{6}\cdot \frac{\text{cell steps}}{\text{second}}\right)$			Speedup	
		Naïve	SoA	SIMD	$\frac{\text{SIMD}}{\text{naïve}}$	$\frac{\text{SIMD}}{\text{SoA}}$
Oakbridge	TP06	2.917	3.039	15.070	5.2	5.0
Oakbridge	JT21	3.622	3.559	17.970	5.0	5.0
Oakbridge	GPB	2.039	2.047	8.204	4.0	4.0
Oakforest	TP06	0.499	0.525	4.081	8.2	7.8
Oakforest	JT21	0.645	0.651	5.116	7.9	7.9
Oakforest	GPB	0.351	0.406	3.526	10.0	8.7
Milan	TP06	1.186	1.336	5.910	5.0	4.4
Milan	JT21	1.590	1.624	6.712	4.2	4.1
Milan	GPB	1.094	1.103	4.073	3.7	3.7
Wisteria	TP06	0.578	0.694	6.604	11.4	9.5
Wisteria	JT21	0.532	0.871	7.649	14.4	8.8
Wisteria	GPB	0.413	0.475	4.211	10.2	8.9
ThunderX2	TP06	0.911	0.951	1.509	1.7	1.6
ThunderX2	JT21	1.629	1.080	1.849	1.1	1.7
ThunderX2	GPB	0.747	0.787	1.179	1.6	1.5

**TABLE 7:** Multi-threaded performance of naïve and auto-vectorised implementations; The FE scheme is used; *C* = 11688851 cells. “SoA” refers to the “struct of arrays” memory layout discussed in Section 2.3.2.

System	Model	Throughput $\left(10^{6}\cdot \frac{\text{cell steps}}{\text{second}}\right)$			Speedup	
		Naïve	SoA	SIMD	$\frac{\text{SIMD}}{\text{naïve}}$	$\frac{\text{SIMD}}{\text{SoA}}$
Oakbridge	TP06	127.8	134.0	481.7	3.8	3.6
Oakbridge	JT21	148.8	147.2	444.7	3.0	3.0
Oakbridge	GPB	87.4	91.1	239.4	2.7	2.6
Oakforest	TP06	55.7	59.8	398.7	7.2	6.7
Oakforest	JT21	68.3	66.1	376.6	5.5	5.7
Oakforest	GPB	36.4	39.5	234.2	6.4	5.9
Milan	TP06	199.4	219.8	920.8	4.6	4.2
Milan	JT21	250.4	256.0	833.5	3.3	3.3
Milan	GPB	170.2	164.5	518.9	3.0	3.2
Wisteria	TP06	27.8	33.2	296.6	10.7	8.9
Wisteria	JT21	25.1	41.0	321.0	12.8	7.8
Wisteria	GPB	19.5	22.4	167.5	8.6	7.5
ThunderX2	TP06	94.1	97.6	137.1	1.5	1.4
ThunderX2	JT21	142.5	114.2	169.3	1.2	1.5
ThunderX2	GPB	75.5	83.1	109.1	1.4	1.3

Table 8 shows the multi-threaded performance in an ensemble simulation with the JT21 model using the FE and GRL1 schemes. The three loop-structures discussed in Section 2.3.4 are shown in separate columns, and we also report the performance when vectorisation is disabled. When the Cell–Time loop structure is successfully auto-vectorised, it outperforms the other two loop structures, as we observe for Oakbridge and Oakforest. Both of the other loop structures are successfully auto-vectorised on all systems, but the Cell–Time–Cell loop structure is more cache friendly and performs better than the Time–Cell loop structure.

**TABLE 8:** Multi-threaded performance of an ensemble simulation using the JT21 model; *C* = 11688851 cells. The most performant implementation for each system is in boldface.

System	SIMD	Cell–Time		Time–Cell		Cell–Time–Cell	
		FE	GRL1	FE	GRL1	FE	GRL1
Oakbridge	On	**631.1**	**358.9**	154.3	132.2	375.2	257.5
Oakbridge	Off	150.5	90.0	85.3	57.8	110.1	71.1
Oakforest	On	**422.4**	**237.6**	108.2	89.1	114.1	94.7
Oakforest	Off	63.5	37.8	16.1	13.7	16.0	14.1
Milan	On	271.8	161.2	292.1	275.2	**729.3**	**499.0**
Milan	Off	269.2	158.7	151.2	105.9	167.4	116.8
Wisteria	On	41.4	19.4	104.5	70.3	**125.1**	**79.1**
Wisteria	Off	40.8	19.6	17.8	12.0	17.5	11.9
ThunderX2	On	103.5	58.6	83.5	60.9	**112.2**	**76.6**
ThunderX2	Off	111.4	62.6	64.1	40.6	79.6	47.9

To quantify the error of the vectorised code relative to the scalar code, we use the relative root-mean-square (RRMS) norm given by

$${\left\|e\right\|}_{\text{RRMS}}=\sqrt{\frac{\sum_{i=1}^{N}{\left(v_{i}-v_{i}^{\text{ref}}\right)}^{2}}{\sum_{i=1}^{N}{\left(v_{i}^{\text{ref}}\right)}^{2}}},$$ (11)

where *N* is the number of time steps. We have solved the TP06 model for one second using a time step Δ*t* = 1 µs for the FE, RL and GRL1 schemes. The RRMS error for the transmembrane potential is reported in Table 9. The RL and GRL1 schemes exhibit a larger error when (exp(x)-1) is used instead of expm1(x).

**TABLE 9:** RRMS error when solving the TP06 model using SIMD and vectorised math functions. For each ODE solver scheme a reference solution is computed on Milan using scalar math functions and with compiler optimisations disabled. The model is solved for 1 s with a time step Δ*t* = 1 µs.

System	FE	RL	GRL1
Oakbridge (with expm1)	2.11 × 10^–16^	1.34 × 10^–16^	9.47 × 10^–17^
Oakbridge	2.00 × 10^–16^	1.48 × 10^–14^	1.29 × 10^–12^
Oakforest	3.40 × 10^–16^	2.40 × 10^–11^	1.17 × 10^–11^
Milan	2.67 × 10^–16^	2.48 × 10^–14^	1.07 × 10^–12^
Wisteria	2.33 × 10^–16^	2.49 × 10^–14^	8.43 × 10^–13^
ThunderX2	1.85 × 10^–16^	2.48 × 10^–14^	7.94 × 10^–13^

### 3.5 Speed and Accuracy Related to Using Lookup Tables

Table 10 reports the multi-threaded performance of naïve, auto-vectorised and lookup table (LUT) implementations for the TP06 model. Across all systems the LUT implementation is 3.5–4.7 times faster than naïve. ThunderX2 clearly favours LUT, which we attribute to its 128-bit vector length. Oakforest and Wisteria, on the other hand, clearly favour SIMD with their 512-bit vector length. On Oakbridge, which also has 512-bit vector units, LUT is marginally faster than SIMD. The fact that SIMD doesn’t perform better on Oakbridge is likely due to the somewhat low speedup of the vectorised math library (see Table 4). On Milan, SIMD and LUT perform very similarly, which is roughly in line with expectations.

**TABLE 10:** Multi-threaded performance of naïve, auto-vectorised and LUT implementations for the TP06 model in a simulation scenario. The Rush–Larsen scheme is used with Δ*t* = 0.1 µs, *C* = 11688851 cells. “SoA” refers to the “struct of arrays” memory layout discussed in Section 2.3.2. The most performant implementation is in boldface.

System	Throughput $\left(10^{6}\cdot \frac{\text{cell steps}}{\text{second}}\right)$					Speedup		
	Naïve	SoA	SIMD	LUT	SIMD & LUT	$\frac{\text{SIMD}}{\text{Naïve}}$	$\frac{\text{LUT}}{\text{Naïve}}$	$\frac{\text{SIMD \& LUT}}{\text{Naïve}}$
Oakbridge	96.0	96.3	374.3	393.4	**552.4**	3.9	4.1	5.8
Oakforest	46.9	47.8	354.3	196.3	**423.5**	7.6	4.2	9.0
Milan	163.2	170.9	754.5	762.3	**1198.0**	4.6	4.7	7.3
Wisteria	17.5	21.3	**245.0**	74.3	206.5	14.0	4.2	11.8
ThunderX2	71.8	79.7	116.0	**250.1**	199.3	1.6	3.5	2.8

When solving the non-gating equations with SIMD vectorisation and the gating equations with LUT, we see an improvement over only SIMD or LUT on the three x86-based systems. As ThunderX2 has a very limited speedup from the use of SIMD vectorisation, the combination of SIMD and LUT performs worse than the LUT implementation. On Wisteria, the use of SIMD vectorisation leads to much greater speedups than the use of LUT, and shifting parts of the computation from SIMD to LUT leads to a loss in performance compared to the pure SIMD variant.

Whereas the speedup of the LUT implementation is largely insensitive to the CPU vector width and depends more on the model formulation, the speedup of the SIMD implementation strongly depends on the vector width. For the TP06 model, it seems that we need more than 4 SIMD lanes for the SIMD implementation to outperform the LUT implementation. However, the observations we have made regarding the speedup solving the TP06 model with a LUT does not generalise to all cell models, and other models may see smaller or larger gains from using a LUT.

**FIGURE 1:**
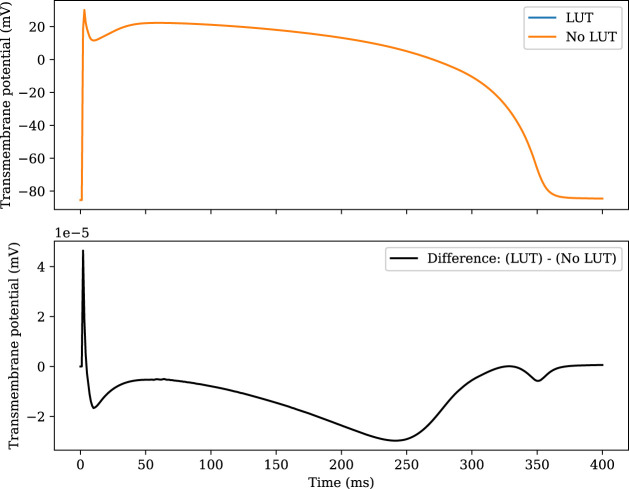
Comparison of the TP06 model solved with and without the use of lookup tables. The RL scheme is used with Δ*t* = 1 µs. In the upper plot, the two numerical solutions cannot be distinguished by eye. The RRMS error of the LUT solution compared with the non-LUT solution is 1.36 × 10^–7^.

Figure 1 compares the accuracy of the LUT implementation to the naïve implementation. The two solutions are plotted together in the top panel, and the difference is plotted in the bottom panel. The error introduced by the LUT is greatest during the upstroke, but the absolute error does not exceed 5 × 10^−5^mV at any point.

### 3.6 Speeding up a 10^9^-Ensemble Computation

Recent studies [see e.g., Tveito et al. (2018); Jæger et al. (2020, 2021a)] have used cardiac cell models to decode the observed effect of a drug on a chip of human induced pluripotent stem cell-derived cardiomyocytes. In these studies, the assumption is that there is a set of model parameters corresponding to the drug effect, and the computational problem consists of searching through the higher-dimensional parameter space. The optimisation problem is particularly expensive because one has to solve the cell model for a long time period until steady state is reached.

In this section, we have set up an ensemble simulation where we try to optimise 11 parameters by pre-computing solutions for a cartesian grid in the parameter space. Note that we treat the remaining model parameters as constant, similar to how all parameters are essentially constant in the simulation scenario. With 5–12 grid points for each parameter, the total number of parameter sets was 1,020,937,500. This parameter mesh can be used directly to solve the optimisation problem by taking the parameter mesh point that minimises the cost function as the solution, or it can be used to guide the search of another optimisation algorithm.

**FIGURE 2:**
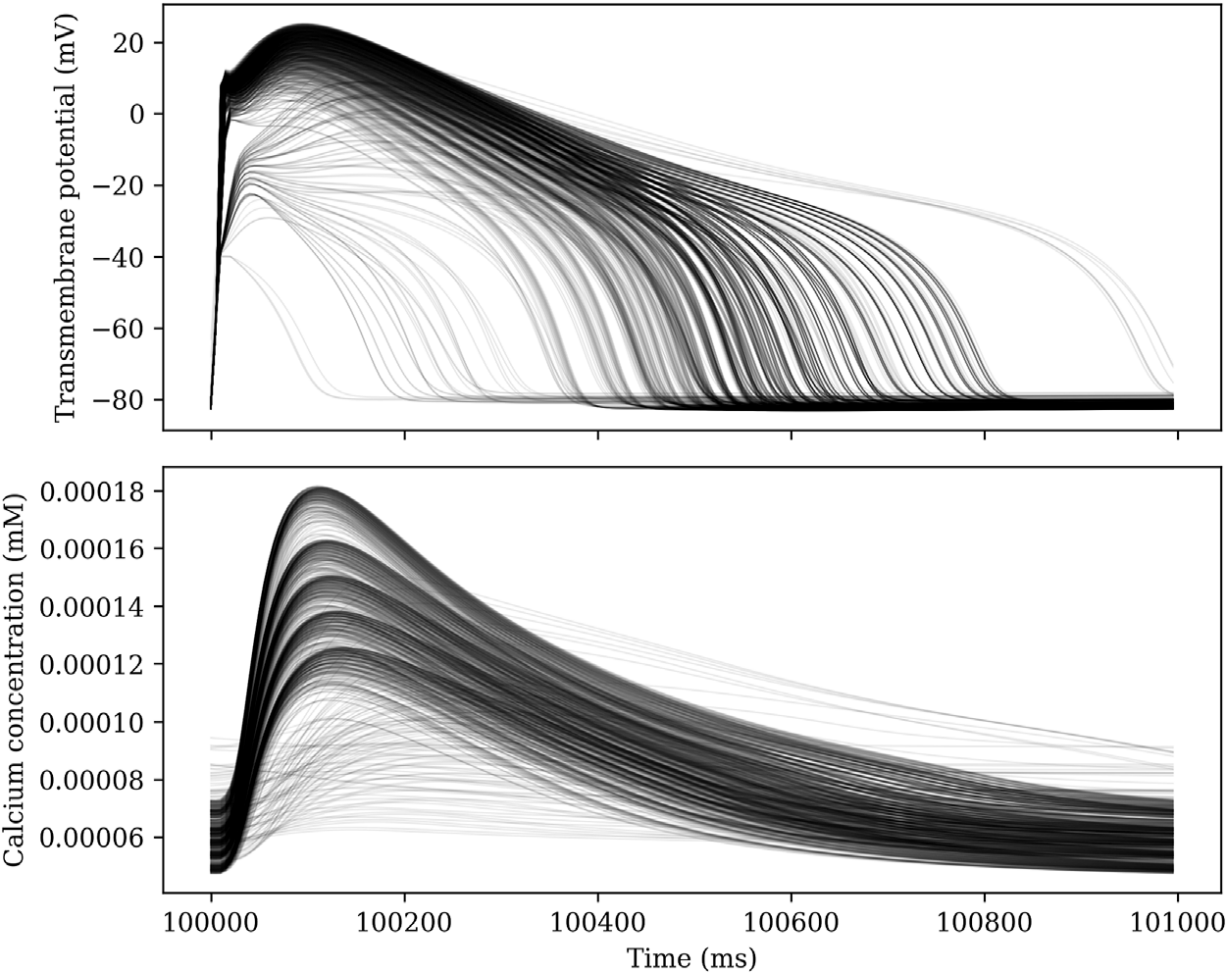
Traces of the transmembrane potential and calcium concentration for 1000 different sets of parameters in an ensemble simulation.

Each parameter set is solved for 102 seconds using a GRL1 scheme with a time step Δ*t* = 10 µs. The model is paced at 1 Hz, and the 100 first seconds are intended to allow the model to reach steady state. We then record the solution for the last 2 seconds at a temporal resolution of 5 ms. By comparing the two last action potentials, we can determine whether steady state has indeed been reached. For some choices of parameters, there is no steady state solution with a period equal to the pacing period. Figure 2 shows traces of the transmembrane potential for 1000 different sets of parameters for *t* ∈ [100 s, 101 s).

The 1,020,937,500 parameter sets were divided into batches of size 50,048 so that we could use a batch job in the queue system on Oakforest to achieve task-level parallelism. Each of the 20,400 batches was solved on a single node, and the resource limits on Oakforest allowed us to use up to 2048 nodes concurrently. This job expended 11,990 tokens (and one token equals an hour of compute time for a single node). We ran one of the batches both with and without SIMD vectorisation so that we can extrapolate an estimate for how many tokens we would have needed if we had run the entire job with a scalar implementation. The codes differ only in the absence of vectorisation compiler hints for the non-SIMD version, i.e. the line #pragma omp for simd is replaced by #pragma omp for. The expm1 function was used to evaluate expressions on the form (*e* ^*x*^ − 1). Without SIMD, the solver achieved a throughput of 37.0 million cell steps per second, whereas the SIMD version reached 232.25 million cell steps per second, equating to a speedup by a factor of 6.28. If we extrapolate from this speedup factor, we can estimate the cost of running the whole simulation without vectorisation to 75,260 node hours. In other words, the use of vectorisation lead to a reduction in compute time of approximately 63,270 node hours. Since the power draw per compute node on Oakforest is approximately 331 W [see TOP500 (2021)], 63,270 node hours translates to an energy consumption of approximately 20,942 kW h.

## 4 Related Work and Conclusion

### 4.1 Related Work

The code vectorisation in this work is automatically enabled by the compilers, with the help of a few hints that are provided in the form of compiler directives and additional clauses. Such an implicit vectorisation approach is programmer-friendly and portable, except perhaps a few #if defined (XXX) directives to accommodate compiler-specific details. The downside is that concerns over safety or efficiency may prevent the compiler from vectorising more complicated code, such as a loop body containing scattered memory accesses due to the use of lookup tables. To handle such situations will require explicit vectorisation. The first alternative is to directly program with SIMD intrinsics. The challenge is that different processor architectures may support different intrinsic instruction sets. For example, AMD CPUs currently only support (extended) SSE instructions with 128-bit and 256-bit vector widths [see AMD64 Technology (2021)], whereas high-end Xeon and Xeon Phi processors also support AVX-512 instructions with 512-bit vector width [see Intel (2021)]. On Arm processors, the diversity is even larger with respect to SIMD vectorisation. There are currently three SIMD instruction set architectures applicable: SVE, Neon and Helium [see Arm Intrinsics (2022)]. However, a specific Arm processor may only support one of them. An explicit vectorisation of the ten Tusscher–Panfilov model for IBM A2 CPUs was developed as part of the “Cardioid” monodomain simulator [see Mirin et al. (2012)]. As a second approach to explicit vectorisation, there are high-level wrapper libraries that offer portability and improved programmability. Two such examples are VCL [see Fog (2017; 2022)] and MIPP [see Cassagne et al. (2018); MIPP (2021)]. Both are implemented using C++ and support various SSE and AVX/AVX-512 instructions, whereas the latter also supports Arm Neon instructions. The single-instruction-multiple-thread (SIMT) execution model found on graphics processing units (GPUs), which resembles CPU-based SIMD execution in some respects, has been applied to cardiac cell models [see e.g., Neic et al. (2012); Sachetto Oliveira et al. (2018)].

Using lookup tables is a widely used approach to saving the computational cost of directly evaluating mathematical functions. The different scientific domains that have used this performance enhancing strategy are summarised in a recent publication [see Marsh et al. (2021)], which also discusses a methodology for predicting the speedup due to using lookup tables. For cardiac simulations in particular, the topic of using lookup tables has been addressed in e.g. Cooper et al. (2006); Mirin et al. (2012); Green et al. (2019), where the latter contains a detailed study about the accuracy loss caused by lookup tables.

### 4.2 Conclusion

We have seen that the largest performance improvement of the ODE solvers arises from using SIMD, and the code vectorisation in this work has been automatically enabled by the compilers. There are two conditions for this “easy” approach. First, some restructurings of a naïve implementation are needed. The most important code restructuring is to re-organise the overall data structure as a “struct of arrays” with padding, see Section 2.3.2. The other code restructurings include swapping the cell–time loop ordering or adding an additional loop level for the ensemble scenario, see Section 2.3.4. Second, appropriate compiler hints are needed inside the source code. We have chosen to use the OpenMP simd construct together with the #pragma omp parallel for directive, as illustrated in Listings 4, 5, 6 and 7. This choice has the benefit of simultaneously enabling SIMD vectorisation and multi-threaded parallelisation, both are essential for achieving the full potential of multi-core CPUs for the ODE solving procedure.

In connection with the compiler-enabled auto-vectorisation, we have presented the necessary compiler options for the different compilers, see Table 3. We have also studied the performance gain (or even loss) due to vectorisation of four frequently used mathematical functions on five hardware testbeds (see Table 4), as well as the appropriate vectorised math libraries to be used. We have found that the relative benefit of using SIMD is correlated with the peak SIMD floating-point throughput of the hardware platform. Moreover, the minor accuracy loss due to using vectorised math libraries can be found in Table 5. It has been shown through actual ODE computations, see Table 9, that the slightly inaccurate vectorised math libraries will not affect the overall accuracy.

The use of lookup tables may interfere with the auto-vectorisation, but we have demonstrated that the two techniques can be used to solve different parts of the cell model, which on some of the target platforms yielded higher performance than using either technique by itself. The decision about whether to use lookup tables is hardware specific. On platforms that only support small vector widths or have no high-quality vectorised math libraries, the speedup potential due to lookup tables can be large. The exact performance benefit, however, depends on the size and resolution of the lookup tables, which may affect the computational accuracy. A direction for future work is to investigate whether explicit vectorisation (using high-level wrapper libraries) can be used to combine SIMD parallelism with lookup tables without partitioning the state variables. In particular, such a combination should be attempted with the help of automated code generation, e.g., inside the modern cardiac simulator openCARP [see Plank et al. (2021); openCARP (2022)].

## Data Availability

The original contributions presented in the study are included in the article/Supplementary Material, further inquiries can be directed to the corresponding author.
